# Correction: Name use by companion parrots

**DOI:** 10.1371/journal.pone.0349699

**Published:** 2026-05-20

**Authors:** The PLOS One Staff

The ORCID iDs are missing for the third, fourth, and fifth author. Please see the authors’ respective ORCID iDs here:

- Author Marisa Hoeschele’s ORCID iD is: 0000-0002-2102-0882
- (https://orcid.org/0000-0002-2102-0882).
- Author Eva Reinisch’s ORCID iD is: 0000-0002-1400-5473
- (https://orcid.org/0000-0002-1400-5473).
- Author Christine R. Dahlin’s ORCID iD is: 0000-0002-8301-519X
- (https://orcid.org/0000-0002-8301-519X).

The publisher apologizes for the error.
